# Experimental Implementation of the Secondary 3 Program of Project P.A.T.H.S.: Observations Based on the Co-Walker Scheme

**DOI:** 10.1100/tsw.2009.118

**Published:** 2009-10-01

**Authors:** Daniel T. L. Shek, Rachel C. F. Sun, Christina Y. P. Tang

**Affiliations:** ^1^ Department of Applied Social Sciences, Hong Kong Polytechnic University, Hong Kong, China; ^2^ Department of Sociology, East China Normal University, Shanghai, China; ^3^ Kiang Wu Nursing College of Macau, Macau, China; ^4^ Department of Social Work, Chinese University of Hong Kong, Hong Kong, China

**Keywords:** quality of curriculum delivery, observation, positive youth development program, process evaluation, program adherence

## Abstract

Classroom observations based on the Co-Walker Scheme were conducted in 34 schools in order to examine the implementation quality of the Tier 1 Program (Secondary 3 Program) of the Project P.A.T.H.S. (Positive Adolescent Training through Holistic Social Programmes) in the Experimental Implementation Phase. Results showed that the overall level of program adherence was generally high (with an average of 82.8%) and the mean ratings of the 13 items examining the implementation quality were all on the high side. Student participation and involvement, and the degree of achievement of the objectives, were the two significant predictors of both overall implementation quality and success of implementation, whereas lesson preparation was the third significant predictor of overall implementation quality. In conjunction with other process evaluation findings, the present study supports that the implementation quality of the Project P.A.T.H.S. is good.

### INTRODUCTION

The project entitled “P.A.T.H.S. to Adulthood: A Jockey Club Youth Enhancement Scheme” is a holistic positive youth development program developed for junior secondary school students in Hong Kong, with “P.A.T.H.S.” denoting **P**ositive **A**dolescent **T**raining through **H**olistic **S**ocial Programmes. There are two tiers of program (Tier 1 and Tier 2) in this project. The Tier 1 Program is a universal positive youth development program for students from Secondary 1 to 3 (Grade 7 to 9 in North America), with the curricula developed by the research team comprised of scholars from different disciplines (e.g., social work, psychology, and education). The Tier 2 Program is a selective program that targets adolescents with greater psychosocial needs and was developed by nongovernmental organizations (NGOs) rendering school social work service.

Weissberg et al.[1] outlined five phases that are critical to the establishment of effective school-based social competence promotion programs:

1. *Conceptualization* stresses the explicit articulation of the program objectives, and the theoretical considerations that guide the selection and formulation of intervention strategies.
2. *Design* involves the construction of developmentally and culturally appropriate training materials and activities, with clearly specified and replicable guidelines for implementation.
3. *Implementation* concerns adaptation of the programs to the ecology of the school setting and the development of measures to ensure the program integrity.
4. *Evaluation* highlights the assessment of the program implementation quality, the effects of training on students, and program variables leading to students' positive changes (or no change).
5. *Maintenance* and *Dissemination* identify program practices and system-level policies or structures that allow beneficial programs to endure and be replicated successfully in potential new sites.

The Tier 1 Program of the Project P.A.T.H.S. is developed in line with the five phases proposed by Weissberg et al.[1]. Regarding conceptualization, this program was developed by integrating existing research findings, researchers' experiences in program construction, local adolescent needs, and cultural characteristics. It basically anchors to 15 positive youth development constructs that have been found to be intrinsic to the existing successful positive youth development programs[2]. Concerning program design, it is a comprehensive and holistic teaching package with clearly specified rationales, implementation guidelines, and lesson plans. In dealing with the major developmental aspects of youth, it covers five ecological dimensions of individual, family, peer, school, and society. Apart from classroom discussions and activities, other means, such as take-home worksheets and small group projects, are used to create spaces for students to learn and develop through experiential learning.

For the implementation phase, systematic and adequate training of 20 h is provided to teachers and social workers to supplement knowledge and skills in order to ensure proper implementation. In addition, flexibility is given to the participating schools in choosing the program implementers (either school teachers or social workers), implementation mode (either 20-h full program or 10-h core program), teaching sequence of units, and the degree of incorporating the program into the formal curriculum. Apart from providing structured and detailed curriculum and teacher training before program implementation, Weissberg et al.[1] also point out that it is equally important to provide classroom assistance and on-site coaching to the participating schools for effective implementation practices. Therefore, the research team launched a "Co-Walker Scheme" in the first year of the Full Implementation Phase, with the aim of understanding the program implementation in schools and providing ongoing support and guidance to the participating schools. In this scheme, each participating school has one assigned co-walker, who is a colleague of the project. The co-walkers are responsible for taking care of the participating schools in the format of providing consultation and support, school visits, and classroom observations. Immediately after each classroom observation, the co-walkers are required to complete a rating form for administrative record. Meetings or discussions with the school personnel are usually held before or after the classroom observation, with the purpose to ensure the quality of the program implementation in schools, to offer support and guidance with respect to the views and needs of the implementers, and to enhance the morale of the front-line workers. With such strong theoretical underpinnings, well-structured program design, and support provided to schools, it is expected that the program will be delivered as designed and benefit the students in the long run.

Regarding the fourth stage mentioned above (i.e., evaluation), numerous objective and subjective outcome evaluation studies[3,4,5,6,7,8] have been conducted to demonstrate the effectiveness of the Tier 1 Program of Project P.A.T.H.S. Moreover, process evaluation, in the format of systematic classroom observation[9,10,11] and interim evaluation[12,13,14], has been carried out. The results clearly showed that program adherence was high, and most of the instructors and students had positive perceptions of the program. Furthermore, the findings of the case study[15] indicated that five groups of factors related to program, people, process, policy, and place contributed to the success of program implementation in schools. Based on these evaluation findings, it is hoped that factors contributing to successful program implementation could be identified and the program could be integrated into the school formal curriculum in the long run. In addition to proper documentation of evaluation findings, sharing sessions on good practices have been organized regularly to school principals, teachers, and social workers, so as to help lead to the fifth phase of maintenance and dissemination proposed by Weissberg et al.[1].

Among the five phases of an effective program, Lane and Beebe-Frankenberger[16] highlight the importance of conducting process evaluation by arguing that "if we cannot be sure of the relationship between the intervention or treatment procedure and the target behavior (internal validity), then we cannot say with confidence that this type of intervention should be effective with other students with similar types of problems (external validity)" (p.131). In addition, without systematic process evaluation findings, researchers would fail to draw valid and accurate conclusions about program outcomes due to the possibility of a Type III error (i.e., existence or nonexistence of program effect because of the occurrence of activities different from those intended by the program developers). In short, it is important to conduct process evaluation in order to understand the nature of program implementation at the school level because it not only links up with program outcomes, but also helps to identify practices and system-level support that allow for maintenance and dissemination of the program.

In the Project P.A.T.H.S., besides process evaluation in the format of systematic classroom observation[9,10,11] and interim evaluation[12,13,14], classroom observations conducted by co-walkers can also provide additional information on the nature of program implementation in schools. In view of the positive findings from the co-walkers' observation conducted in the first 2 years of the Experimental Implementation Phase (EIP-S2: 2006/07)[17] and Full Implementation Phase (FIP-S1 and S2: 2007/08)[18,19], it is expected that the participating schools in the third year of the Experimental Implementation Phase will deliver the Tier 1 Program (Secondary 3 Program) as intended and in high quality because of having accumulated experiences.

Nevertheless, there are some factors that may influence the implementation process at the Secondary 3 level. First, it is the education policy that may affect the weight attached to positive youth development programs in schools. Following the traditional secondary education system, Secondary 3 students in the school year of 2007/08 were still under the Junior Secondary Education Assessment (JSEA) for the allocation of Secondary 4 places. As the priority of Secondary 4 place allocation would be based on students' school examination results, teachers, students, and their parents might place more concern on academic achievement, and less emphasis on other learning, such as resilience training. The second factor refers to the characteristics of Secondary 3 students. Compared to their Secondary 1 and 2 counterparts, Secondary 3 students were found to have poorer mental health, including existential well-being, sense of mastery, life satisfaction, and general health[20,21]; poorer parent-child relational qualities[21]; and lower levels of perceived family functioning[22]. Therefore, regarding the unique learning climate and students' development, it is interesting to utilize the data of the co-walkers' observation to examine (1) the program implementation quality and adherence of the Tier 1 Program in the Secondary 3 level, (2) the differences in the quality of program delivery between the Secondary 2 level and Secondary 3 level of the Project P.A.T.H.S. in the Experimental Implementation Phase, and (3) the predictors of overall implementation quality and implementation success.

### METHODS

#### Participants

Among the 48 schools that joined the Secondary 3 Program of the Project P.A.T.H.S. in the Experimental Implementation Phase in the school year 2007/08, 19 adopted the 20-h full program that involves 40 teaching units and 29 adopted the 10-h core program that involves 20 teaching units. All participating schools were invited to join the present study, but only 34 schools allowed the research team to conduct classroom observations. Among these schools, 12 schools adopted the full program and 22 schools adopted the core program. The average number of students per class was 35.64 and the average number of instructors per class was 1.70. As each teaching unit was designed to be a 30-min lesson, the average duration of observation of one teaching unit was 37.55 min.

### Procedures and Instrument

In each school that joined the present study, systematic observations of one or two teaching units were conducted. There were 44 units under observation, which covered 14 positive youth development constructs, including bonding, social competence, emotional competence, cognitive competence, behavioral competence, moral competence, self-efficacy, prosocial norms, resilience, self-determination, spirituality, clear and positive identity, beliefs in the future, and prosocial involvement.

The observers were seven co-walkers who were colleagues of the project, six of whom were registered social workers and one who had a doctoral degree. During the observations, each co-walker observed how the units were implemented and was required to complete an observation form covering four major areas, including basic information, implementation mode of units, program fidelity and adherence, and quality of program delivery. For program fidelity and adherence, the co-walker rated the degree of adherence in percentage and recorded the time used to implement the unit. For the quality of program delivery, 13 items, including student interest, student participation and involvement, classroom control, use of interactive delivery method, use of strategies to enhance student motivation, use of positive and supportive feedback, instructors' familiarity with the students, opportunity for reflection, degree of achievement of the objectives, time management, quality of preparation, overall implementation quality, and success of implementation, were rated on a 7-point scale.

## RESULTS

Table 1 shows the implementation mode of each observed unit. Among 44 observed units, 61.4% (i.e., 27 units) were incorporated into the formal curriculum, such as Liberal Studies or Religious Studies, while 31.8% (i.e., 14 units) were implemented outside the formal curriculum, such as postexamination days or class teachers' periods. For the remaining units, 4.5% (i.e., two units) were implemented in another time slot (e.g., borrowed lessons from other subjects) and 2.3% (i.e., one unit) were partly incorporated into the formal curriculum of the Life Education subject and partly implemented in class teachers' periods.

**TABLE 1**
**The Distribution of Observed Tier 1 Program Units Implemented in Different Modes for Schools Adopting 10 and 20 Hours of Implementation**

| Different Mode | Hours of Implementation | | |
|---|---|---|---|
| | 10 | 20 | Total |
| Incorporated into the formal curriculum | 18 (64.3%) | 9 (56.3%) | 27 (61.4%) |
| Outside formal curriculum | 10 (35.7%) | 4 (25.0%) | 14 (31.8%) |
| Other time slot | 0 | 2 (12.5%) | 2 (4.5%) |
| Incorporated into formal curriculum and outside formal curriculum | 0 | 1 (6.3%) | 1 (2.3%) |
| Total | 28 (100%) | 16 (100%) | 44 (100%) |

*Note*: Outside formal curriculum refers to the implementation after school, during holidays, in class teachers' periods, postexam days, assemblies, or camps.

As shown in Table 2, the average overall adherence to the curriculum manual was 82.84% (range: 30–100%), which was generally high. For those units where modifications had been made, the observers regarded them as reasonable. The quality of implementation of the Tier 1 Program (Secondary 3 Program) was also good. As shown in Table 2, the mean ratings of the 13 items examining the quality of program delivery were all over 4.5 on a 7-point rating scale, with the highest rating of 5.02 on lesson preparation, and the lowest rating of 4.55 on the use of positive and supportive feedback. Reliability analysis showed that the scale was internally consistent (α = 0.94; mean interitem correlation = 0.57).

**TABLE 2**
**Average Rates of Quality of Curriculum Delivery and Overall Adherence in Secondary 2 and 3 Programs of the Experimental Implementation Phase**

| Quality of Curriculum Delivery | Secondary 2 (2006/07) (n = 15) | Secondary 3 (2007/08) (n = 44) | t-Value |
|---|---|---|---|
| | M (SD) | M (SD) | |
| 1. Student interest | 5.33 (1.35) | 4.82 (0.90) | 1.68 |
| 2. Student participation and involvement | 5.40 (1.35) | 4.84 (0.94) | 1.77 |
| 3. Classroom control | 5.47 (1.13) | 4.57 (1.09) | 2.74** |
| 4. Interactive delivery method | 5.40 (1.06) | 4.70 (1.13) | 2.09* |
| 5. Strategies to enhance student motivation | 5.33 (0.82) | 4.57 (1.17) | 2.34* |
| 6. Use of positive and supportive feedback | 5.33 (1.18) | 4.55 (0.95) | 2.61** |
| 7. Instructors' familiarity with the students | 5.07 (1.39) | 4.80 (1.46) | 0.63 |
| 8. Opportunity for reflection | 4.80 (1.08) | 4.57 (1.02) | 0.75 |
| 9. Degree of achievement of the objectives | 5.53 (1.13) | 4.64 (1.06) | 2.79** |
| 10. Time management | 5.20 (1.37) | 4.61 (1.24) | 1.54 |
| 11. Lesson preparation | 5.93 (0.96) | 5.02 (1.13) | 2.79** |
| 12. Overall implementation quality | 5.60 (1.06) | 4.68 (1.07) | 2.87** |
| 13. Success of implementation | 5.40 (0.99) | 4.59 (0.97) | 2.77** |
| **Overall Adherence (%)** | 81.87 | 82.84 | — |

*Note*: $^{**}p < 0.01$, $^{*}p < 0.05$.

Although the overall quality of program implementation was good, results of independent-samples t-test showed that the mean ratings of most of the items of the curriculum delivery quality of the Secondary 3 Program were significantly lower than those of the Secondary 2 Program (EIP-S2: 2006/07)[17], such as in the aspects of overall implementation quality, degree of achievement of the objectives, lesson preparation, success of implementation, classroom control, use of positive and supportive feedback, use of strategies to enhance student motivation, and use of interactive delivery method (see Table 2).

As shown in Table 3, the 13 items examining the curriculum delivery were positively intercorrelated. Specially, the overall implementation quality (item 12) and success of implementation (item 13) were highly correlated (r = 0.92, $p < 0.01$). Moreover, both were significantly and positively correlated with all the other items, and had relatively high correlations with student participation and involvement (item 2, r = 0.73 and r = 0.74, $p < 0.01$), classroom control (item 3, r = 0.76 and r = 0.73, $p < 0.01$), degree of achievement of the objectives (item 9, r = 0.82 and r = 0.85, $p < 0.01$), and lesson preparation (item 11, r = 0.73 and r = 0.64, $p < 0.01$).

**TABLE 3**
**Intercorrelations among Items of the Curriculum Delivery Assessment**

| Item | 1 | 2 | 3 | 4 | 5 | 6 | 7 | 8 | 9 | 10 | 11 | 12 | 13 |
|---|---|---|---|---|---|---|---|---|---|---|---|---|---|
| 1. Student interest | 1.00 | | | | | | | | | | | | |
| 2. Student participation and involvement | 0.74** | 1.00 | | | | | | | | | | | |
| 3. Classroom control | 0.61** | 0.64** | 1.00 | | | | | | | | | | |
| 4. Interactive delivery method | 0.56** | 0.59** | 0.57** | 1.00 | | | | | | | | | |
| 5. Strategies to enhance student motivation | 0.48** | 0.57** | 0.56** | 0.78** | 1.00 | | | | | | | | |
| 6. Use of positive and supportive feedback | 0.45** | 0.52** | 0.44* | 0.48** | 0.61** | 1.00 | | | | | | | |
| 7. Instructors' familiarity with the students | 0.49** | 0.42** | 0.52** | 0.56** | 0.37* | 0.39** | 1.00 | | | | | | |
| 8. Opportunity for reflection | 0.32* | 0.53** | 0.46** | 0.49** | 0.54** | 0.66** | 0.46** | 1.00 | | | | | |
| 9. Degree of achievement of the objectives | 062** | 0.60** | 0.65** | 0.61** | 0.58** | 0.53** | 0.48** | 0.71** | 1.00 | | | | |
| 10. Time management | 0.29 | 0.48** | 0.61** | 0.33* | 0.33* | 0.36* | 0.21 | 0.62** | 0.67** | 1.00 | | | |
| 11. Lesson preparation | 0.51** | 0.44** | 0.63** | 0.55** | 0.54** | 0.49** | 0.64** | 0.65** | 0.69** | 0.52** | 1.00 | | |
| 12. Overall implementation quality | 0.62** | 0.73** | 0.76** | 0.63** | 0.69** | 0.58** | 0.45** | 0.66** | 0.82** | 0.66** | 0.73** | 1.00 | |
| 13. Success of implementation | 0.63** | 0.74** | 0.73** | 0.61** | 0.64** | 0.47** | 0.38* | 0.66** | 0.85** | 0.66** | 0.64** | 0.92** | 1.00 |

*Note*: **$p < 0.01$, *$p < 0.05$.

Based on these findings of correlations, separate multiple regression analyses were performed to examine the predictive contribution of the 11 aspects of program delivery on (1) overall implementation quality and (2) success of implementation. Results in Table 4 show that the overall implementation quality was significantly predicted by student preparation and involvement ($\beta = 0.38$, $p < 0.01$), degree of achievement of the objectives ($\beta = 0.39$, $p < 0.01$), and lesson preparation ($\beta = 0.34$, $p < 0.01$). The model explained for 86% of the variance in overall implementation quality [$F$ (11, 43) = 17.91, $p < 0.01$]. Similarly, success of implementation was significantly predicted by student preparation and involvement ($\beta = 0.32$, $p < 0.05$) and degree of achievement of the objectives ($\beta = 0.49$, $p < 0.01$). The model explained for 86% of the variance in implementation success [$F$ (11, 43) = 17.45, $p < 0.01$].

### DISCUSSION

The main purpose of this paper was to examine the implementation of the Tier 1 Program (Secondary 3 Program) in the aspects of adherence and quality of delivery. Several observations could be highlighted from the present findings. First, consistent with the previous classroom observation findings obtained in both the Experimental Implementation Phase (EIP-S1: 2005/06 and EIP-S2: 2006/07)[9,10] and Full Implementation Phase (FIP-S1: 2006/07)[11], high program adherence rate was found. The present findings clearly suggest that no major modifications on the Secondary 3 Program of the Tier 1 Program are needed. Although an acceptable degree of variation was allowed to accommodate individual schools' needs, it might make the program susceptible to undesirable modifications that are inconsistent with the original program framework, resulting in reduced program fidelity. In order to ensure that the Tier 1

**TABLE 4**
**Summary of the Multiple Regression Analyses**

| Overall Implementation Quality | | Success of Implementation | |
|---|---|---|---|
| **Significant Predictors** | **β** | **Significant Predictors** | **β** |
| 1. Student participation and involvement | 0.38** | 1. Student participation and involvement | 0.32* |
| 2. Degree of achievement of the objectives | 0.39** | 2. Degree of achievement of the objectives | 0.49** |
| 3. Lesson preparation | 0.34** | | |
| | $R^2 = 0.86$ | | $R^2 = 0.86$ |
| | $F(11, 43) = 17.91$** | | $F(11, 43) = 17.45$** |

*Note*: **$p < 0.01$, *$p < 0.05$.

Program is properly delivered across schools, the co-walkers truly serve as important agents to maintain the communication between program developers and program implementers, and to discuss vital vs. adaptable program components with the implementers. In short, as high program adherence is a crucial element contributing to an effective program, provision of on-site guidance and support is equally important to guarantee program fidelity and delivery quality in the implementation phase[1].

Second, in the aspect of program delivery, the implementation quality observed in the present study was regarded as satisfactory. In line with the co-walkers' observation of the Tier 1 Program in the Experimental Implementation Phase (2006/07 school year)[17] and Full Implementation Phase (2007/08 school year)[18,19], high appreciation was particularly paid to the implementers' effort in preparing the lessons. It reflects that the implementers were dedicated and motivated to implement the program well. The motivation of the program implementers might be attributed to their perceptions that the Tier 1 Program was positive and beneficial to students[12,13,14]. Both their devotion and positive perception of the program might be attributed to the 3-day training workshop provided for instructors before the implementation, from which they developed a sense of ownership of the program and mission to promote positive youth development.

However, as reflected from the present and previous findings[9,10,11], it seems that the program implementers were less competent to offer positive and supportive feedback to their students, provide opportunity for students' reflection, enhance student motivation, and maintain classroom discipline. Since these skills were found to play crucial roles in creating a positive classroom atmosphere that facilitates students' learning[15], the present findings throw light on the importance of mentioning such implementation skills in the instructors' training workshops, in order to equip the implementers with necessary skills (e.g., interactive skills, self-reflection) to achieve program success. Furthermore, since Weissberg et al.[1] indicate that having opportunities to demonstrate teaching and classroom management techniques is valuable in enhancing teachers' program delivery skills, it would be fascinating if the experienced and skilled implementers could be invited to train other workers in the training workshops, to share their experiences in briefing sessions, or even to allow site visits of their program delivery.

Third, another area that deserved attention was the declining trend of program implementation quality in the Secondary 3 level. One possible reason might be due to the Junior Secondary Education Assessment (JSEA), which leads the schools to place a stronger emphasis on academic learning rather than on positive youth development. As school policy is an important factor facilitating or impeding the implementation process[15], it is illuminating to examine further how the school policies, such as having explicit goals of student development, integration of the youth development program into the school's formal curriculum, and good manpower deployment, might contribute to successful implementation of the Tier 1 Program in the Secondary 3 level. However, it should be noted that the present findings might not clearly reflect the actual situation because the comparisons were based on two different groups of schools in the school years of 2006/07 and 2007/08, and the sample size was relatively small. Furthermore, different co-walkers involved in the rating may also contribute to the observed differences.

Finally, the findings showed that there were high intercorrelations among the items in the curriculum delivery assessment. Multiple regression analyses revealed that student participation and involvement, and the degree of achievement of the objectives, were the two significant predictors of both overall implementation quality and success of implementation, whereas lesson preparation was the third significant predictor of overall implementation quality. This observation was generally consistent with the previous findings[19] that higher levels of student involvement and lesson preparation significantly contributed to higher levels of program delivery quality and success. These findings also demonstrated that "people" was a vital process variable leading to successful implementation[15]. On one hand, students need to have motivation to join classroom activities, and on the other hand, instructors need to be responsible to prepare the lessons well. Furthermore, the present findings based on observations of the Secondary 3 Program added that accomplishing the learning objectives of each unit was another critical determinant contributing to implementation quality and success. It echoes the views of Fagan and Mihalic[23] on "fidelity" to the intended program as the key process variable of quality implementation.

There are several limitations of the present study. First, since only one observer was involved in the classroom observation, there might be biases that affect the credibility of the findings. Second, consistent with the intrinsic problem of all observational studies where time sampling is involved, one needs to be conscious of the degree of generalizability of the present findings to other temporal and spatial contexts. Possible confounding factors are that the students may become more cooperative and the instructors may be more motivated to teach well when there are visitors and outside observers. Therefore, the use of ethnographic strategies with prolonged engagement and observations would be helpful. Third, the differences in program implementation quality between the Secondary 2 and 3 Programs should be interpreted with caution as cohort and observer differences may account for the findings. Despite these limitations, the present findings document the integrity of program implementation through the evaluation of process variables, and support that the Tier 1 Program of the Project P.A.T.H.S. has been implemented successfully in schools.

### ACKNOWLEDGMENTS

The preparation for this paper and the Project P.A.T.H.S. were financially supported by The Hong Kong Jockey Club Charities Trust.
